# Limonene formulation exhibited potential application in the control of mycelial growth and deoxynivalenol production in *Fusarium graminearum*

**DOI:** 10.3389/fmicb.2023.1161244

**Published:** 2023-03-23

**Authors:** Yunqing Jian, Xia Chen, Haiqin Ma, Changpeng Zhang, Yuqin Luo, Jinhua Jiang, Yanni Yin

**Affiliations:** ^1^State Key Laboratory for Managing Biotic and Chemical Threats to the Quality and Safety of Agro-products, Key Laboratory for Pesticide Residue Detection of Ministry of Agriculture, Institute of Agro-Product Safety and Nutrition, Zhejiang Academy of Agricultural Sciences, Hangzhou, Zhejiang, China; ^2^State Key Laboratory of Rice Biology, Institute of Biotechnology, Zhejiang University, Hangzhou, Zhejiang, China; ^3^Oro Agri International Ltd, Fresno, CA, United States

**Keywords:** limonene formulation, *Fusarium graminearum*, antifungal activity, antitoxic effect, DON production

## Abstract

Preventing grain from fungi and subsequent mycotoxins contamination has attracted notable attention. Present study demonstrated the limonene-formulated product Wetcit^®^, might be a biocontrol agent and potential alternative to synthetic fungicides to control *Fusarium graminearum* growth and deoxynivalenol (DON) production. The limonene formulation exhibited antifungal activity against *F. graminearum* with the EC_50_ at 1.40 μl/ml, electron microscopy and staining analysis showed limonene formulation could significantly decrease the quantity, length and septa of conidia, caused hyphal break and shrink, damaged the structures of cell membrane, cell wall, vacuoles and organelles in the hypha. Further study revealed the antifungal and antitoxic mechanism of limonene formulation against *F. graminearum*, limonene formulation significantly inhibited the toxisome and DON formation, was associated with the down-regulation of trichothecenes biosynthesis genes expression and many energy metabolism pathways as well as the inhibition of lipid droplets, the disturbed energy homeostasis and intracellular structures might ultimately inhibit fungal growth and DON production. In addition, limonene formulation enhanced the antifungal activity of triazole fungicides tebuconazole and mefentrifluconazole against *F. graminearum*, indicated limonene formulation has valuable potential as a bio-alternative fungicide and eco-friendly compound preparation for the effective management of *F. graminearum* and DON contamination in agriculture.

## Introduction

Preventing food products from fungal infection and mycotoxin contamination have aroused broad public concerns as food security is of particular importance to human and animal health globally (Mishra et al., 2020). The trichothecenes, zearalenones and fumonisins as the common mycotoxins in cereals, have attracted widespread attention for their considerable toxicity and widely existing in food and feed products (Chen et al., 2019; Mishra et al., 2020). The type B trichothecene mycotoxin deoxynivalenol (DON), is found the most prevalent and frequently detected in cereals under field and storage conditions worldwide (Lee and Ryu, 2017; Chen et al., 2019). It was found the incidence rate of DON in cereal grains ranged from 50% in Asia to 76% in Africa, and had a higher occurrence in feedstuffs with 75% average incidence rate in corn samples from Central Europe and North America (Streit et al., 2013; Lee and Ryu, 2017). DON can inhibit protein translation by binding to peptidyl transferase protein in the ribosome, then causes emetic effects, immune dysregulation, reproductive and teratogenic effects in mammals (Rocha et al., 2005; Lu et al., 2022). DON remains stable during storage and is resistant to thermal processing (Sugita-Konishi et al., 2006), once the agricultural commodities are contaminated with DON, it will exhibit great health hazard to animals and humans.

Many mycotoxins can be produced by *Fusarium* spp., the greatest economic losses are associated with DON and its derivatives (Dean et al., 2012). DON is primarily produced by *Fusarium graminearum*, which is the predominant and devastating pathogen of Fusarium head blight (FHB) that has become one of the most significant diseases in cereal crops (Xu and Nicholson, 2009). In China, between 2000 and 2018, the occurrence of FHB can affect exceed 20% of the total wheat planted area, consequently reduces yields and grain quality (Chen et al., 2019). Based on the economic importance of FHB and DON toxicity, *F. graminearum* was considered as the fourth most threatening plant pathogenic fungi (Dean et al., 2012). As the DON contamination in infected agricultural commodities is closely associated with the severity of *F. graminearum* infection in field (Harris et al., 1999; Paranidharan et al., 2008), the effective measure to manage the DON contamination is to decrease *F. graminearum* pathogenicity and control FHB disease during crop cultivation.

Recently, the plant secondary metabolites essential oils are gaining popularity in the agricultural field because of their low toxicity and excellent activity against plant pathogenic fungi (Das et al., 2021; Tian et al., 2021; Cai et al., 2022; Punetha et al., 2022). Many essential oils have been reported that could inhibit the growth of *F. graminearum* and affected the morphology of mycelia and spores, such as the essential oils obtained from *Ocimum sanctum* L., *Curcuma longa* L., with minimum inhibitory concentration at 1,250 and 2,450 μg/ml, respectively (Kalagatur et al., 2015, 2018). Limonene is a monocyclic monoterpene hydrocarbon, which is found in the essential oils from more than 300 plants and principally in *Citrus* spp., and more than 90% of essential oil from citrus is D-limonene (Ravichandran et al., 2018; Mahato et al., 2019). Limonene is commonly used as food preservative, food and cosmetics additive (Ravichandran et al., 2018), also has therapeutic efficacy in medicinal industry (Anandakumar et al., 2021). Furthermore, studies have demonstrated that limonene possess a good application prospect in agriculture field (Ibáñez et al., 2020; Akhavan-Mahdavi et al., 2022). Literatures showed limonene or essential oils containing limonene had inhibitory effects on different fungi, such as *Sclerotinia sclerotiorum*, *Rhizoctonia solani*, *Penicillium digitatum*, *Fusarium* sp. and *Aspergillus* sp. (Marei et al., 2012; Ma et al., 2015; Bulkan et al., 2021; Li et al., 2021; Luchesi et al., 2022), also could simultaneously decrease DON (Kalagatur et al., 2018; Perczak et al., 2019) as well as aflatoxin production in *A. flavus* (Xiang et al., 2020; Li et al., 2021). Therefore, limonene possesses valuable potential as a bio-alternative to synthetic fungicides to control phytopathogenic fungi and mycotoxins.

Literatures demonstrated essential oils containing limonene had antifungal activity on *F. graminearum* (Krzyśko-Łupicka et al., 2019; Luchesi et al., 2022), which was a combination effect caused by limonene and the bioactive phytochemical metabolites present in essential oils. Limited studies have evaluated the effect of limonene commercial products on fungal growth and DON synthesis of *F. graminearum*. So in current study, we evaluated antifungal activity of the commercial product WETCIT^®^ (5% D-limonene SL), which is recommended for use as insecticides, fungicides and herbicides (ORO AGRRI, 2018), but few reports have evaluated its antifungal and antitoxic effect on *F. graminearum* as a candidate fungicide. We determined the interference of limonene formulation on hyphal and conidial morphology, toxisome and DON production, further transcriptional analysis was used to elucidate the antifungal and anti-toxigenic mechanism. Together with the synergistic interactions between limonene formulation and widely used synthetic fungicides, the results could provide insights that limonene can be a novel bio-alternative to synthetic fungicides in the management of FHB and mycotoxin.

## Materials and methods

### Strains and sensitivity tests

The parental strain used in this study is wild-type strain PH-1 (NRRL 31084) of *F. graminearum*. To test the mycelial inhibition by limonene formulation WETCIT^®^ (5% D-limonene SL; Oro Agri International Ltd., Fresno, United States), the PH-1 strain was cultured on potato dextrose agar (PDA; 200 g potato, 20 g glucose, 10 g agar and 1 l water) containing different concentrations of limonene formulation at 25°C for 2 days, then the colony diameter of each plate was measured. The values of effective concentrations that result in 50 and 90% mycelial growth inhibition (EC_50_ and EC_90_), were calculated by the Data Processing System (DPS) program (Hangzhou Reifeng Information Technology Ltd., Hangzhou, China).

### Conidiation and conidial morphology examination

In order to study the impact of limonene formulation on *F. graminearum* conidiation, mycelial plugs of wild-type strain PH-1 were inoculated into a flask containing 30 ml carboxymethylcellulose (CMC) broth (Yun et al., 2015), then incubated at 25°C with 180 rpm. After 24 h of incubation, limonene formulation was added into each flask to generate final concentrations of 0 (control, CK), EC_50_ (1.40 μl/ml) and EC_90_ (14.0 μl/ml). After incubation for another 3 days, the conidia of each treatment were firstly filtered and then determined by hemocytometer. The conidial length was measured with SPOT software, septa were stained with Calcofluor White Stain, and the conidial morphology was examined with a fluorescence microscope. Each experiment was repeated twice, and each concentration included three replicates.

### Electron microscope examination of hyphal morphology changes

The alterations of fungal morphological caused by limonene formulation, were observed with scanning electron microscope (SEM, TM-1000, Hitachi, Japan) and transmission electronic microscopy (TEM, JEM 1230, JEOL, Akishima, Japan). Samples treated with or without limonene formulation (1.40 and 14.0 μl/ml) for 2 h were prepared following the steps. Firstly, the mycelia of each treatment were immersed in glutaraldehyde [2.5% (*v*/*v*)] overnight, and then post-fixed with osmium tetroxide [1% (*w*/*v*)] for 2 h at room temperature. Subsequently, samples were well dehydrated with gradients of ethanol (30–100%). Then the extracellular and intracellular changes were observed by SEM and TEM analysis, respectively.

### Staining and fluorescence microscope observation of mycelia

To analyze possible changes in cell membranes caused by limonene formulation, we stained the cell membrane with a commonly used membrane-selective fluorescent dye FM4-64 (Fischer-Parton et al., 2000). Simply, the treated mycelia were immersed into FM4-64 solution (7.5 μM) for 1 min at room temperature, and immediately observed the stained mycelia under a Zeiss LSM780 confocal microscope (Gottingen, Niedersachsen, Germany).

The vacuole lumenal marker 7-amino-4-chloromethylcoumarin (CMAC) was used to evaluate the influence of limonene formulation on vacuoles. The mycelia of each treatment were stained with 100 μM CMAC solution at room temperature for 30 min, and washed by sterile water for three times. Finally, the stained mycelia were subjected to microscopic analysis using Zeiss LSM780 confocal microscope.

To investigate the effect of limonene formulation on the formation of lipid droplets, the hyphae of the treatment were stained with Nile Red, which is the most commonly used fluorescent dye to quantify neutral lipid content (Kimura et al., 2004). Each sample was stained with Nile Red staining solution, which containing 20 mg/ml polyvinylpyrrolidone and 2.5 mg/ml Nile Red Oxazone (Sigma-Aldrich, HY-D0718) in 50 mM Tris-maleate buffer (pH 7.5). After staining for 2 min, the lipid droplets were observed under Zeiss LSM780 confocal microscope.

### The effect of limonene formulation on DON production

FgTri1 can reflect the toxisome formation and usually correlates with DON levels (Menke et al., 2013). To address the influence of limonene formulation on toxisome formation, *FgTRI1-GFP* fusion cassettes were introduced into ΔFgTri1 mutant to generate ΔFgTri1::FgTri1-GFP strain, which was produced in our previous study (Yun et al., 2014). The mycelia of FgTri1-GFP labeled strain were cultured in Biosynthesis Inducing (TBI) medium for 24 h at 28°C, 150 rpm in the dark, then limonene formulation was added to cultures to final concentrations of 0.0050 μl/ml, 0.050 μl/ml, 0.20 μl/ml, 1.00 μl/ml, 1.40 μl/ml, 2.00 μl/ml for 24 h. The formation of toxisome was indicated by FgTri1-GFP marker and was observed under a Zeiss LSM780 confocal microscope (Gottingen, Niedersachsen, Germany). The FgTri1-GFP protein levels were further detected by western blot assays, and the details of procedures were listed in the supplemental information (SI).

To further determine DON production, the mycelia of PH-1 were cultured in TBI medium for 24 h at 28°C in the dark, then limonene formulation was added to the cultures at 0.050, 0.20, 1.00, 1.40 μl/ml for additional 6 days. The filtrate liquid was collected to quantify DON production using DON Quantification Kit Wis008 (Wise Science, Zhenjiang, China). Then qRT-PCR assays were conducted to evaluate the impact of limonene formulation on the transcription of DON biosynthesis genes. The mycelia of PH-1 were firstly incubated in TBI media at 28°C for 48 h. Then limonene formulation was supplemented to generate final concentrations of 0.200 μl/ml for another 24 h. The treatments without limonene were used as control. The expression levels of six key trichothecenes biosynthesis genes (*FgTRIs*), *FgTRI1*, *FgTRI4*, *FgTRI5*, *FgTRI6*, *FgTRI12* and *FgTRI101*, were determined using HiScript II Q RT SuperMix (Vazyme Biotech, Nanjing, China) by qRT-PCR, and *FgACTIN* gene was used as the endogenous control. The experiments were performed with independent biological triplicates. The RNA extraction and relevant primers to perform qRT-PCR assay were listed in SI and Supplementary Table S1, and the relative expression levels of *TRI* genes were calculated by 2^−ΔΔCt^ formula (Livak and Schmittgen, 2001).

### RNA-sequencing and analysis

To further investigate the antifungal mechanism of limonene formulation against *F. graminearum*, RNA-Sequencing (RNA-Seq) analysis was conducted. PH-1 strain was firstly cultured in yeast extract peptone dextrose (YEPD) medium at 25°C, 180 rpm for 36 h, and then supplemented with 0.20 μl/ml limonene formulation for another 2 h. The limonene formulation untreated mycelia were served as control. Samples were quickly collected and froze in liquid nitrogen immediately, then stored at −80°C for further RNA-Seq. The RNA library construction was conducted according the manufacturer’s procedures, and the details were listed in the SI.

After construct RNA libraries, adapters and low-quality reads were filtered by using the cutadapt (version 1.11). When we mapped the clean reads to the *F. graminearum* reference transcripts using Hisat2 (version 2.1.0), at most two mismatches were allowed during the process. These genes were subjected to alignment against public protein databases; Pfam (Pfam Protein families), Uniprot (Swiss-Prot). It comprised RSEM (v1.2.6) for transcript abundance estimation and normalization of expression values as FPKM (Fragments per kilobase of transcript per million fragments mapped). Differentially expressed genes (DEGs) were identified with DESeq2 with a filter threshold of *p*-value <0.05 and|log_2_FoldChange| > 1. ClusterProfiler^1^ in R package was employed to perform GO and KEGG (Kyoto Encyclopedia of Genes and Genomes, http://www.genome.jp/kegg/) enrichment analysis. The GO and KEGG enrichment analysis were calculated using hypergeometric distribution with a Q value cutoff of 0.05. Q values obtained by Fisher’s exact test were adjusted with FDR for multiple comparisons.

### Synergistic potential of limonene formulation with chemical fungicides against fusarium

To explore whether limonene formulation would increase the control efficacy of chemical fungicides, the synergistic potential of limonene formulation in combination with tebuconazole, mefentrifluconazole and phenamacri against *F. graminearum* and *F. fujikuroi*, were determined using the mycelial growth rate method. The EC_50_ of each fungicide was showed in Supplementary Table S2. Three mixture ratios of limonene formulation and chemical fungicides were used to assess the synergistic interactions, with 1:1, 2:1, 5:1 of limonene formulation and each chemical fungicide, and the concentration of each mixture was 1 mg/ml. Thereafter, the mixtures were diluted with PDA medium to five concentrations, and the diluted concentrations of the mixtures were showed in Supplementary Table S3. To determine the synergistic interactions of the mixtures, Wadley’s model was used to calculate the theoretical EC_50_ (EC_50th_) and the interaction level (R; Tak et al., 2017), details of the calculation of EC_50_ values of the mixture were listed in the SI. The synergistic interaction of mixtures was defined as synergistic when *R* > 1.5, additive when 1.5 > *R* > 0.5, and antagonistic when *R* < 0.5.

## Results

### Effect of limonene formulation on mycelial growth and conidia production

**Figure 1:**
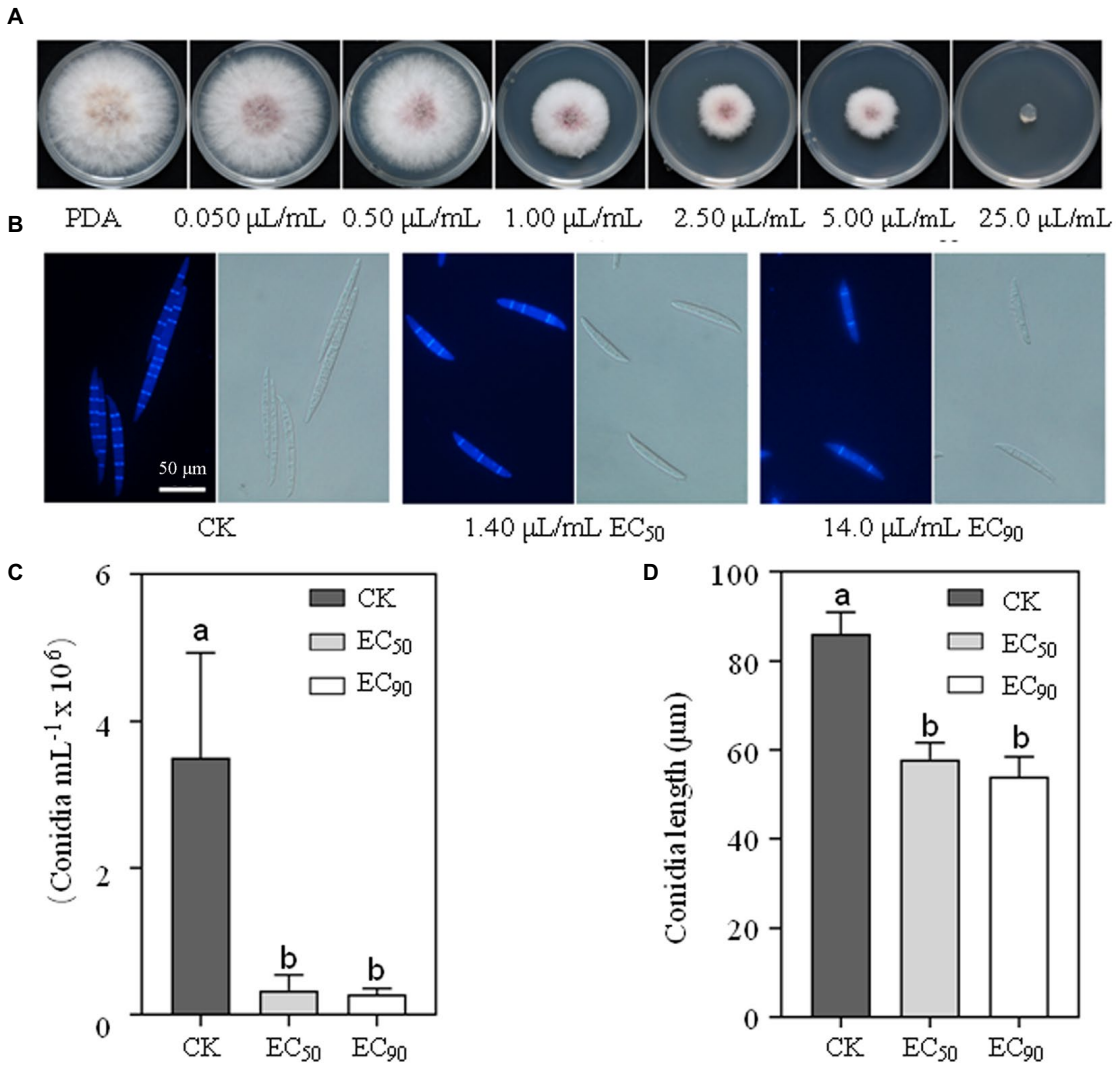
Limonene formulation inhibited the mycelial growth and conidia production of *Fusarium graminearum.* **(A)** Limonene formulation with different concentrations inhibited the mycelial growth of PH-1. **(B)** Limonene formulation caused change in conidial morphology and quantity of PH-1. **(C)** Limonene formulation decreased the numbers of conidia. **(D)** The length of the conidia was reduced by limonene formulation. Line bars in each column denote SE of three repeated experiments. Values on the bars followed by the same letter are not significantly different at *p* = 0.05.

To explore the activity of limonene formulation against *F. graminearum*, we tested its effect on mycelial growth of the wild-type strain PH-1. As shown in Figure 1A, the mycelial growth was inhibited as the limonene formulation concentration increased. The effective concentrations of limonene formulation that inhibit mycelial growth by 50 and 90% relative to the control, i.e., EC_50_ and EC_90_, were calculated as 1.40 μl/ml and 14.0 μl/ml against *F. graminearum*, respectively. Results further showed 1.40 μl/ml and 14.0 μl/ml limonene formulation significantly decreased the numbers of septa (Figure 1B), reduced the quantity and length of conidia (Figures 1C,D).

### The influence on hyphal intracellular and membrane structures

**Figure 2:**
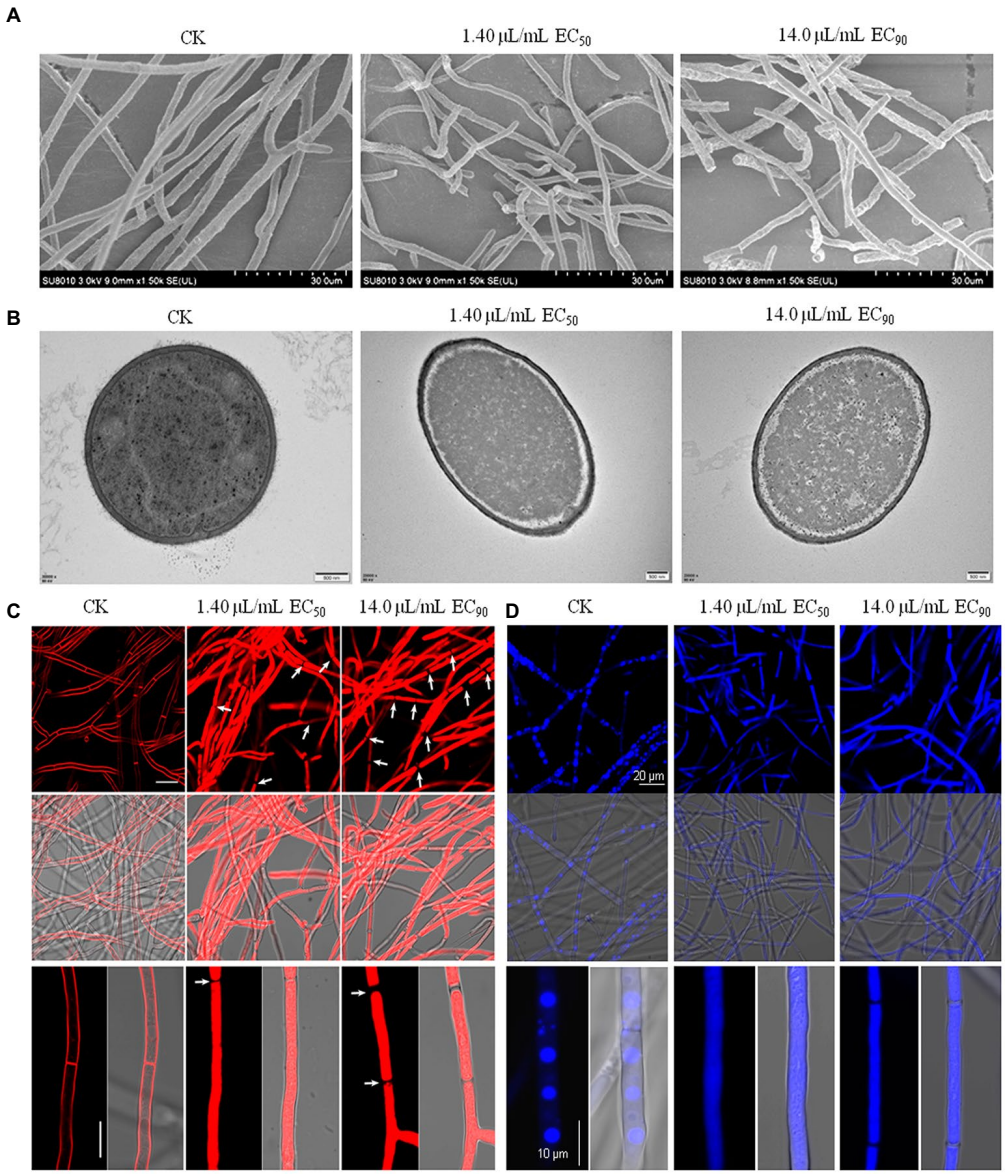
Limonene formulation disrupted hyphal morphology and structures of *Fusarium graminearum*. **(A)** SEM images of PH-1 mycelia treated with or without limonene formulation treatments. Bars, panel = 30 μm. **(B)** TEM images of PH-1 mycelia after treated with limonene formulation. Bars, panel = 500 nm. **(C)** FM4-64 staining analyze the influence of limonene formulation on cell membrane. **(D)** Fluorescence images of the vacuoles in hyphae after staining with CMAC.

SEM and TEM assays were conducted to explore the morphological changes of extra- and intra-cellular fungal cells caused by limonene formulation. SEM showed limonene formulation caused hyphal break and shrink to a certain degree (Figure 2A). TEM assay further showed the hyphal cell membrane clearly detached from the cell wall, and the organelles were seemed to be degraded by limonene formulation, as no obvious organelle was observed as compared to control (Figure 2B).

The mycelia were stained with FM4-64 to observe the influence of limonene formulation on cell membrane. In the control group, the hyphae exhibited intact form, and strong red fluorescent signal were observed at septa and strictly along the cell membrane, while weak signal could be seen in some of the intracellular organelles, such as vacuoles and endosomes (Figure 2C).Whereas, limonene formulation treatment resulted in hyphal break, and extremely bright fluorescence was evenly distributed in the whole hyphae (Figure 2C). CMAC staining further revealed that limonene formulation could cause the abnormality of vacuoles. As shown in Figure 2D, the hypha formed round and clear vacuole lumen in the control group, but normal vacuoles could hardly be observed in those of limonene formulation treated groups, as the blue fluorescent signal was detected in the whole hyphae.

### Inhibition of mycotoxin DON production by limonene formulation

**Figure 3:**
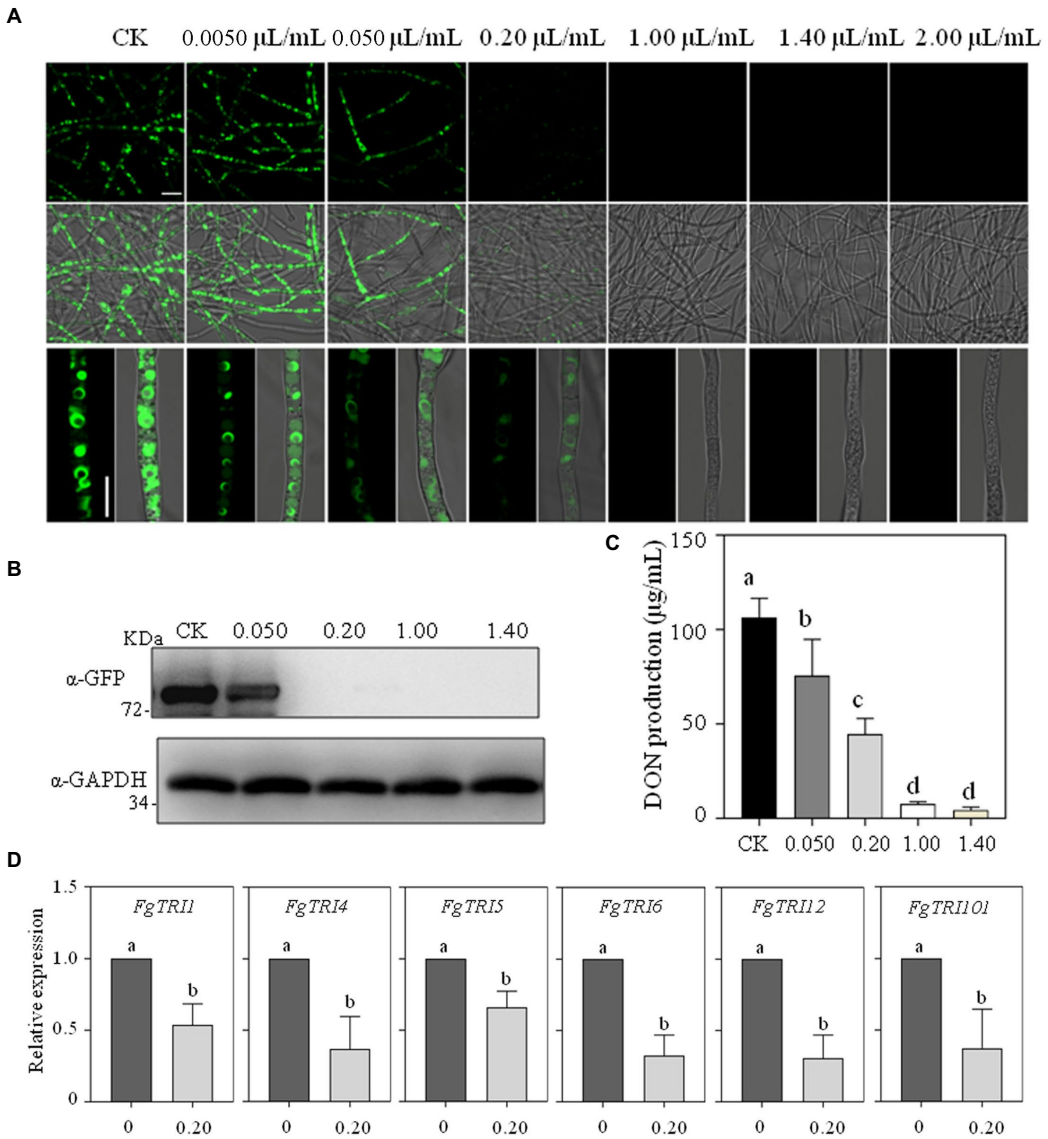
Limonene formulation inhibited toxisome formation and DON production in *Fusarium graminearum.* **(A)** Limonene formulation with different concentrations impeded toxisome formation. The fluorescence signal of ΔFgTri1::FgTri1GFP strain was examined after limonene formulation treatment. Bars, panel = 10 μm. **(B)** FgTri1-GFP protein levels were decreased by limonene formulation. **(C)** Mycotoxin DON production was inhibited by limonene formulation. Different letters represent statistically significant differences according to the one-way ANOVA test (*p* < 0.05). **(D)** The relative expression levels of DON biosynthetic genes were decreased by the treatment of 0.20 μl/ml limonene formulation. Different letters represent statistically significant differences according to the one-way ANOVA test (*p* < 0.05).

Under toxin-inducing condition, toxisomes were formed and green fluorescent signal marked these special spherical structures were observed in the control group (Figure 3A). Results demonstrated that 0.050 μl/ml limonene formulation could significantly reduce the FgTri1-GFP fluorescent signal, only faint FgTri1-GFP signal could be seen in vision in the 0.20 μl/ml treatment (Figure 3A). When the concentrations of limonene formulation further increased, fluorescent signal could no longer be observed (Figure 3A). Western blotting assay further validated that the protein levels of FgTri1-GFP could not been detected in the 0.20 μl/ml, 1.00 μl/ml and 1.40 μl/ml limonene formulation treatments (Figure 3B).

As toxisome formation was usually correlated with DON levels, the impact of limonene formulation on DON production was further evaluated. Results showed that mycotoxin DON production was significantly inhibited by 0.050, 0.20, 1.00, 1.40 μl/ml limonene formulation (Figure 3C). RT-PCR further demonstrated that the transcription level of six key *FgTRI*s involved in trichothecenes biosynthesis, such as *FgTRI1*, *FgTRI4*, *FgTRI5*, *FgTRI6*, *FgTRI12* and *FgTRI101*, was dramatically down-regulated by 0.20 μl/ml limonene formulation (Figure 3D).

### Effect of limonene formulation on transcriptome in *Fusarium graminearum*

**Figure 4:**
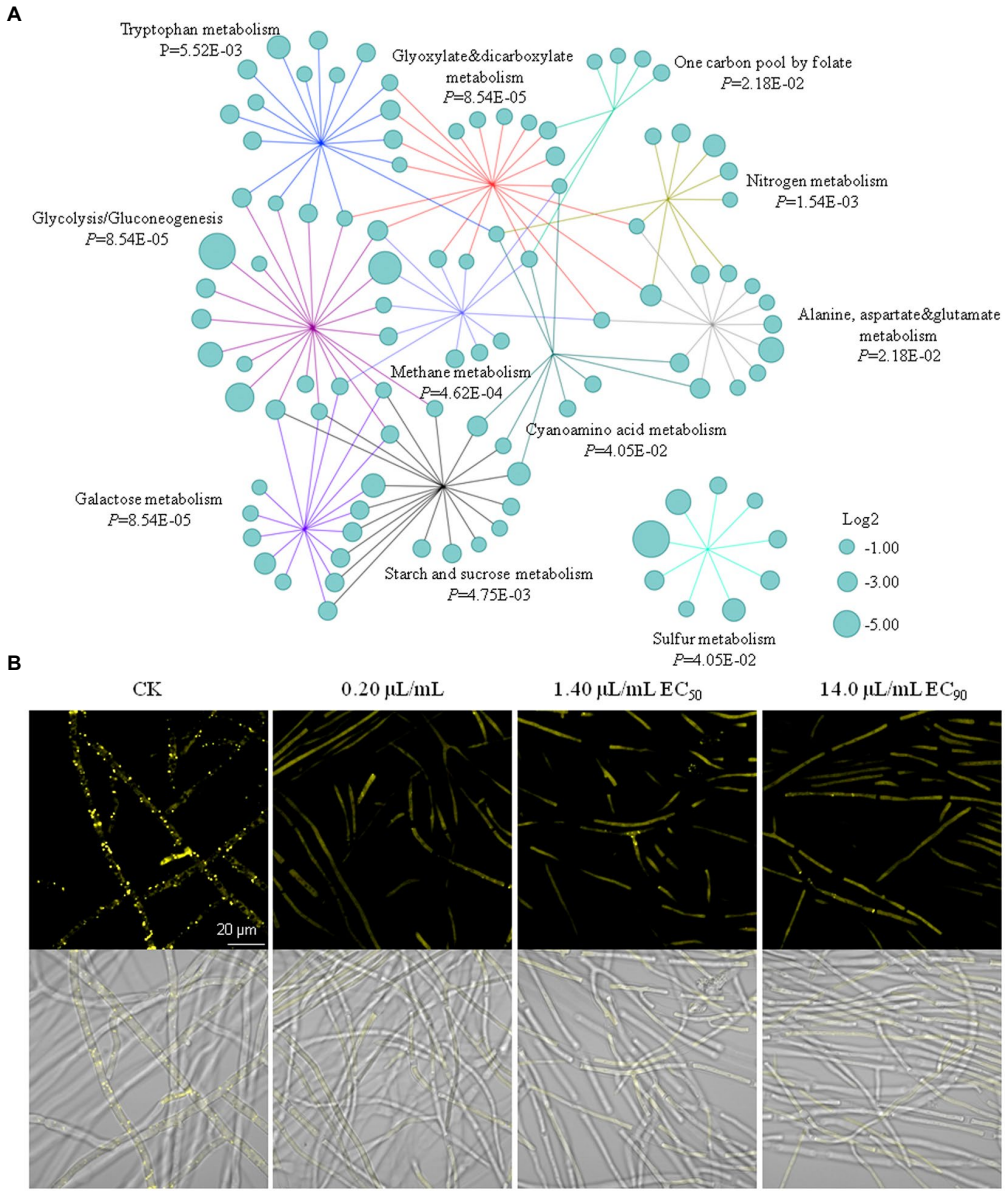
Limonene formulation inhibited the energy metabolism pathways and lipid droplets formation in *Fusarium graminearum*. **(A)** Network analysis of the relationship between significantly down-regulated genes and the corresponding enriched KEGG pathways. The cyan circle indicated different gene. Involvement of these genes in different metabolism pathways was visualized with colored lines representing each pathway. The relative fold change (log2-transformed) of gene expression is indicated by the circle size. **(B)** Limonene formulation inhibited the accumulation of lipid droplets in the hyphae.

A total of 3,657 genes were differentially expressed after 0.20 μl/ml limonene formulation exposure. Among them, 1,723 genes were up-regulated, and 1,934 genes were down-regulated (Supplementary Figure S1). We categorized the DEGs and analyzed by KEGG, Supplementary Figure S2 showed the top 20 enriched KEGG pathways identified in *F. graminearum* after limonene formulation treatment. Strikingly, major metabolism pathways for generating energy and functional proteins were significantly down-regulated, and such as galactose metabolism, glycolysis/gluconeogenesis, glyoxylate dicarboxylate metabolism, amino acids metabolism, methane metabolism, sulfur metabolism and nitrogen metabolism and starch/sucrose metabolism (Supplementary Figure S2; Supplementary Table S4). Network analysis further demonstrated the integrated relationship among the DEGs in energy metabolism pathways (Figure 4A).

In addition, ten significant (*p* adjust < 0.05) pathways were up-regulated by 0.20 μl/ml limonene formulation (Supplementary Table S5; Supplementary Figure S2), and nine out of the ten-up-regulated pathways are associated with nucleus processes, such as ribosome biogenesis in eukaryotes, DNA replication, ribosome, RNA polymerase, mismatch repair, RNA transport, cell cycle and meiosis (Supplementary Figure S3).

### The accumulation of lipid droplets is suppressed

Lipid droplets are storage organelles at the center of lipid and energy homeostasis (Olzmann and Carvalho, 2019). The hyphae were stained with Nile Red to examine the effect of limonene formulation on lipid droplets formation. The results showed the hyphae in the control treatment contained a vast number of yellow dots, which indicated lipid droplets. However, the lipid droplets were barely observed in the 0.20, 1.40, and 14.0 μl/ml limonene formulation treatments (Figure 4B), suggested limonene formulation could inhibit the accumulation of lipid droplets in the hyphae.

### Synergistic potential of limonene formulation in combination with chemical fungicides

To explore the control efficacy of limonene formulation combined with chemical fungicides, the synergistic potential of limonene formulation between tebuconazole, mefentrifluconazole and phenamacri against *F. graminearum* and *F. fujikuroi* were determined. As shown in Supplementary Table S6, limonene formulation exhibited synergistic effects with tebuconazole and mefentrifluconazole against *F. graminearum*, at the mixture ratio of 1:1 and 5:1. While the limonene formulation in combination with tebuconazole and mefentrifluconazole at 2:1 showed additive effect, and the mixtures of limonene formulation and phenamacri showed additive effect against *F. graminearum*. For *F. fujikuroi*, except the mixture of limonene formulation and tebuconazole at 5:1 showed synergistic effect, the other mixtures showed additive effect (Supplementary Table S7).

## Discussion

*Fusarium* infections not only cause enormous yield losses, but also pose serious threat to animal and human health *via* the contamination of grain with various mycotoxins (Dean et al., 2012). The DON, as the toxigenic secondary metabolites of *F. graminearum*, has been globally regarded as the predominant contaminant of food and feed commodities. Therefore, the effective management of *F. graminearum* and DON contamination has become a cause of great concern for the cereal production industry. With the increased popular awareness of environmental protection, plant-derived essential oils have attracted notable attention in the alternatives to the use of synthetic pesticides for control of fungal contamination, as the bioactive compounds are considered to be environmentally friendly, broad-spectrum antifungal effect and low toxicity (Gao et al., 2021; Cai et al., 2022; Punetha et al., 2022). In the present study, the results demonstrated the limonene formulation product Wetcit^®^, of which the bioactive ingredient is essential oil D-limonene from *Citrus* spp., might be a potential novel fungicide to control *F. graminearum* growth and DON production.

Literatures demonstrated essential oils containing limonene had varying degrees of antifungal activities against many phytopathogenic fungi, including *F. graminearum* (Marei et al., 2012; Ma et al., 2015; Bulkan et al., 2021; Li et al., 2021; Luchesi et al., 2022). It was found lemon containing 48.3% D-limonene could completely inhibit the mycelial growth of *F. graminearum* isolates at concentrations of 0.5–2.0% (Krzyśko-Łupicka et al., 2019), the essential oils of *Baccharis dracunculifolia* including 10.5% D-limonene at 8.0 and 4.0 μl/ml showed 57.1 and 49.5% inhibitory effect against *F. graminearum* (Luchesi et al., 2022). Moreover, limonene-containing fruits (pear, orange, grapefruit, lemon) are effective in increasing resistance to *F. graminearum* (Supplementary Figure S4). Current researches showed the antifungal activity mediated by limonene, was interacted synergistically with other bioactive phytochemical metabolites present in natural essential oils, limited studies have evaluated the effect of limonene commercial products on fungal growth and development. Although the commercial product Wetcit^®^ is recommended for use as insecticides, fungicides and herbicides (ORO AGRRI, 2018), there are few reports have evaluated its antifungal and antitoxic effect on *Fusarium* as a candidate fungicide. The obtained results confirmed a high effectiveness of limonene formulation in the inhibition of *F. graminearum* growth, with EC_50_ at 1.40 μl/ml. Besides, it was worth noting that limonene formulation had the potential to inhibit DON synthesis. Therefore, further analysis of the mechanism of limonene formulation on the regulation of fungal development and DON production, would be helpful for its efficient application in disease and toxin control in crops and provide better protection for human and animal health.

Essential oils have multiple target sites to inhibit the growth and activity of fungi, including the cell membrane, cell wall, mitochondria and efflux pumps (Dassanayake et al., 2021). SEM analysis showed limonene formulation caused hyphal break and shrink, TEM and FM4-64 staining further showed the structures of cell membrane, cell wall and organelles in the hypha were damaged after the treatment of limonene formulation. Similar findings were reported in previous studies, the essential oils containing limonene could disrupt the membrane integrity or reduce the cytoplasmic content in *F. avenaceum* and *F. graminearum* (Naveen Kumar et al., 2016; Zhang et al., 2022). Studies have found that the essential oils could penetrate into the cell and alter the cell membrane integrity, further lead to the impairment of membrane fluidity and interfere with cellular metabolism (Naveen Kumar et al., 2016; Dassanayake et al., 2021). It was reported that fungal vacuoles are organelles surrounded by cell membrane, and participate in a variety of crucial functions, such as degradative process, primary storage site, osmoregulation and homeostatic regulation process, like cytosolic ion, basic amino acid concentration and intracellular pH (Klionsky et al., 1990). Current study showed the abnormality of vacuoles caused by limonene formulation, was associated with the down-regulation of many energy metabolism pathways in *F. graminearum*, which might thereby activate the transcription of genes involved in nucleus processes. These results indicated that limonene formulation inhibited mycelial growth by destroying the hyphal cell membrane and cell wall structures in *F. graminearum*, then lead to the leakage of cytoplasmic content and the disturbance of osmotic regulation, ultimately depleted the energy metabolism that are crucial for cell growth, ATP synthesis and proliferation of *F. graminearum*.

The cytochrome P450 oxygenases Tri1 catalyzes the late step in trichothecene biosynthesis, co-localizes to vesicles designated “toxisomes,” which can be stimulated under DON inducing conditions (Menke et al., 2013). Hence, FgTri1 can reflect toxisome formation and usually correlates with DON levels (Menke et al., 2013; Chen et al., 2019). Current study found limonene formulation could inhibit the formation of DON and toxisome at the same time, as the FgTri1-GFP fluorescent signal and protein level was significantly reduced in the 0.200, 1.00, and 1.40 μl/ml treatments. Investigators have revealed essential oils containing limonene inhibited the zearalenone and DON production in *F. graminearum*, which was resulted from the reduction of fungal biomass and ergosterol production (Naveen Kumar et al., 2016; Ferreira et al., 2018; Kalagatur et al., 2018). In current study, the results showed the DON synthesis was also correlated with the transcription of trichothecenes biosynthesis genes, the *FgTRI1*, *FgTRI4*, *FgTRI5*, *FgTRI6*, *FgTRI12* and *FgTRI101* expression level was dramatically decreased by limonene formulation. Furthermore, lipid droplets are storage organelles and can associate with most cellular organelles through membrane contact sites, the dynamic of lipid droplets modulates the coordination between different organelles and acts as a vital hub for cellular metabolism (Olzmann and Carvalho, 2019). In current study, limonene formulation significantly down-regulated many energy metabolism pathways, simultaneously inhibited the accumulation of lipid droplets and accompanying the damage of cell membrane and organelles, which might lead to energy imbalance and DON reduction. In addition, the conidiation play important roles in the initial infection of *F. graminearum* (Jenczmionka et al., 2003). Results demonstrated limonene formulation reduced the conidial production and altered the morphology of conidia, suggested limonene formulation might first interfere with the *F. graminearum* infection, damaged the cell structures then disturbed the lipid and energy homeostasis, ultimately impeded the *F. graminearum* hyphal development and DON synthesis.

Currently, the fastest and most effective method for suppressing FHB is still the application of chemical fungicides, such as demethylation inhibitor fungicides prothioconazole, tebuconazole and difenoconazole, the novel myosin inhibitor phenamacril widely used in China, which can effectively control FHB and DON production (Paul et al., 2008; Dweba et al., 2017; Chen et al., 2019). Nevertheless, frequent and excessive use of fungicides promotes the development of pesticide resistance and reduce the control efficiency, also lead to environmental, ecotoxicological and health hazards (Dweba et al., 2017; Chen et al., 2019; Zhang et al., 2020; Man et al., 2021). Many reports have found the botanical essential oils are capable of enhancing the antifungal activity of synthetic fungicides, and can be used as sustainable and natural candidates to control fungi (Dassanayake et al., 2021). Studies have elucidated that combination of the essential oils with azoles had synergistic potential against fungi, such as the *Citrus aurantium* essential oil enhanced the antifungal efficacy of fluconazole (Nidhi et al., 2020), essential oil from *Tagetes filifolia* exhibited synergistic potential with difenoconazole against the phytopathogenic fungus (Gadban et al., 2020). Current study demonstrated limonene formulation potentiated the fungicidal activity of triazole fungicides tebuconazole and mefentrifluconazole when used in 1:1 and 5:1 combination against *F. graminearum*, and exhibited additive effect with phenamacri. Besides, limonene formulation also exhibited antifungal effect, had additive or synergistic effect in the combination with fungicides against the *Fusarium* genus *F. fujikuroi*, which further indicated the limonene formulation as a natural candidate to control *Fusarium*. Moreover, the Wetcit^®^ is also a surfactant distributed by Oro Agri, it was found surfactant Wetcit^®^ and glyphosate formulation mixes significantly decreased the predatory activity of spiders (Niedobová et al., 2019), Wetcit^®^ exhibited synergistic effect when combined with imidacloprid, and simultaneously increased the maximum retention and deposition amount on leaves (Wang et al., 2019). Current research findings indicated the combined application of limonene formulation and triazole fungicides could interact synergistically to mediate the antifungal activity against *Fusarium*, the limonene formulation can be applied to develop eco-friendly compound preparation with extensive application prospects in agrochemical formulations.

In summary, the obtained results confirmed a high effectiveness of the limonene -formulated product Wetcit^®^ in the inhibiting of *F. graminearum* growth and DON production. The disturbance of osmotic regulation and energy metabolism caused by limonene formulation, might ultimately impede the fungal growth and DON synthesis. The synergistic interactions between limonene formulation and triazole fungicides, further extend the application of limonene formulation as a valuable source of pharmaceutical formulation in the alternatives to the use of synthetic pesticides for control of *F. graminearum* in agriculture.

## Data availability statement

The datasets presented in this study can be found in online repositories. The names of the repository/repositories and accession number(s) can be found at: https://www.ncbi.nlm.nih.gov/-, PRJNA937318.

## Author contributions

YJ and XC were involved in methodology, experiments, draft and critical revision of article, and final approval. HM was involved in conceptualization and resources. CZ and YL were involved in data acquisition and data consulting. YY conceived and designed the manuscript. JJ was involved in funding acquisition and writing—review and editing. All authors contributed to the article and approved the submitted version.

## Funding

The present study was supported by grants from the National Natural Science Foundation of China (32272577), the State Key Laboratory for Managing Biotic and Chemical Threats to the Quality and Safety of Agro-products (2021DG700024-KF202203), Fundamental Research Funds for the Central Universities (2021FZZX001-31), Precision training of Zhejiang Academy of Agricultural Sciences. National Key Research and Development Program of China (2022YFD1700500)” here, right before “Fundamental Research Funds for the Central Universities.

## Conflict of interest

The authors declare that the research was conducted in the absence of any commercial or financial relationships that could be construed as a potential conflict of interest.

## Publisher’s note

All claims expressed in this article are solely those of the authors and do not necessarily represent those of their affiliated organizations, or those of the publisher, the editors and the reviewers. Any product that may be evaluated in this article, or claim that may be made by its manufacturer, is not guaranteed or endorsed by the publisher.

## Footnotes

^1^http://www.bioconductor.org/packages/release/bioc/html/clusterProfiler.html
