# Supplementary material for: Limonene formulation exhibited potential application in the control of mycelial growth and deoxynivalenol production in Fusarium graminearum
Source: Front Microbiol. 2023 Mar 23;14:1161244. doi: 10.3389/fmicb.2023.1161244 (PMC10131186; doi:10.3389/fmicb.2023.1161244)
Supplement: Supplementary file 1 [file Data_Sheet_1.docx]

**Supplemental Information (SI)**

**1. Materials and methods**

The effect of limonene formulation on DON production

RNA-Sequencing and analysis

Synergistic potential of limonene formulation with chemical fungicides against *Fusarium*

**2. Supplementary tables**

**Table S1** The primer and information of the gene used in the RT-PCR.

**Table S2** The EC_50_ of limonene formulation, tebuconazole, mefentrifluconazole and phenamacril against *F. graminearum* and *F. fujikuroi* (mg/L).

**Table S3** The detailed of the ratio and concentration gradient of limonene formulation in combination with chemical fungicides against *Fusarium*.

**Table S4** The top 20 pathways and DEGs in the down-regulated KEGG identified in *F. graminearum* (*P*-value <0.05).

**Table S5** The pathways and DEGs in the up-regulated KEGG identified in *F. graminearum* (*P*-value <0.05).

**Table S6** The synergistic potential of limonene formulation in combination with chemical fungicides against *F. graminearum*.

**Table S7** The synergistic potential of limonene formulation in combination with chemical fungicides against *F. fujikuroi*.

**3. Supplementary figures**

**FIGURE S1** The differentially expressed genes caused by 0.20 μL/mL limonene formulation in *F.graminearum*.

**FIGURE S2** The top 20 enriched KEGG pathways identified in *F. graminearum* after 0.20 μL/mL limonene formulation treatment.

**FIGURE S3** Network analysis of the relationship between significantly up-regulated genes and corresponding KEGG pathways induced by limonene formulation.

**FIGURE S4** D-limonene-containing fruits were effective in increasing resistance to *F.graminearum*.

**1. Materials and methods**

*The effect of limonene formulation on DON production*

FgTri1 can reflect the toxisome formation and usually correlates with DON levels (Menke et al., 2013). To address the influence of limonene formulation on toxisome formation, *FgTRI1-GFP* fusion cassettes were introduced into ΔFgTri1 mutant to generate ΔFgTri1::FgTri1-GFP strain, which was produced in our previous study (Yun et al., 2014). The mycelia of FgTri1-GFP labeled strain were cultured in Biosynthesis Inducing (TBI) medium for 24 h at 28°C, 150 rpm in the dark, then limonene formulation was added to cultures to final concentrations of 0.0050 μL/mL, 0.050 μL/mL, 0.20 μL/mL, 1.00 μL/mL, 1.40 μL/mL, 2.00 μL/mL for 24 h. The formation of toxisome was indicated by FgTri1-GFP marker and was observed under a Zeiss LSM780 confocal microscope (Gottingen, Niedersachsen, Germany). The FgTri1-GFP protein levels were further detected by western blot assays. Briefly, about 500 mg mycelia of each treatment were grounded in liquid nitrogen and suspended in 1 mL of protein extraction buffer containing 10 μL proteinase inhibitor (Sangon, Shanghai, China). After homogenization, protein samples were then centrifuged in 4°C at 10, 000 *g* for 15 min. The resulting supernatants were detected by western blot assay with monoclonal anti-GFP (ab32146, Abcam, Cambridge, UK) antibody, and monoclonal anti-GAPDH antibody (EM1101, Hua An Biotech. Ltd., Hangzhou, China, 1:5000 dilution) were indicated as a reference antibody. Each experiment was conducted for three times.

Then qRT-PCR assays were conducted to evaluate the impact of limonene formulation on the transcription of DON biosynthesis genes. The mycelia of PH-1 were firstly incubated in TBI media at 28°C for 48 h. Then limonene formulation was supplemented to generate final concentrations of 0.200 μL/mL for another 24 h. The treatments without limonene were used as control. After that, the mycelia of each treatment were harvested and quickly froze in liquid nitrogen. TRIzol (TaKaRa Biotechnology, Dalian, China) reagent test kit was used for total RNA extraction according to the manufacturer’s instructions. In order to detect the transcription of each gene, the cDNA transcripts were synthesed by using a HiScript II 1^st^ Strand cDNA Synthesis Kit (Vazyme Biotech, Nanjing, China). Further, the expression levels of six key trichothecenes biosynthesis genes, *FgTRI1*, *FgTRI4*, *FgTRI5*, *FgTRI6*, *FgTRI12*, and *FgTRI101,* were determined using HiScript II Q RT SuperMix (Vazyme Biotech, Nanjing, China) by qRT-PCR, and *FgACTIN* gene was used as the endogenous control. The experiments were performed with independent biological triplicates. The RNA extraction and relevant primers to perform qRT-PCR assay were listed in SI and Table S1, and the relative expression levels of *TRI* genes were calculated by 2^−ΔΔCt^ formula (Livak and Schmittgen, 2001).

*RNA-Sequencing and analysis*

To further investigate the antifungal mechanism of limonene formulation against *F. graminearum*, RNA-Sequencing (RNA-Seq) analysis was conducted. PH-1 strain was firstly cultured in yeast extract peptone dextrose (YEPD) medium at 25°C, 180 rpm for 36 h, and then supplemented with 0.20 μL/mL limonene formulation for another 2 h. The limonene formulation untreated mycelia were served as control. Samples were quickly collected and froze in liquid nitrogen immediately, then stored at -80°C for further RNA-Seq.

Following the manufacturer’s instructions, total RNA of each treatment was extracted by using the RNA prep Pure Kit DP432 (TIANGEN Biotech Co.,Ltd., Beijing, China). Before RNA library construction, the integrity of all RNA samples were firstly assessed using Qsep1 instrument. About 3 μg of total RNA was used to construct RNA libraries with the MGIEasy mRNA Library Prep Kit. During the process, polyA-selected RNA extraction, RNA fragmentation, random hexamer primed reverse transcription, and 100 nt paired-end sequencing by MGI 2000 were included in the procedure.

Adapters and low-quality reads were filtered by using cutadapt (version 1.11). When we mapped the clean reads to the *F. graminearum* reference transcripts using Hisat2 (version 2.1.0), at most two mismatches were allowed during the process. These genes were subjected to alignment against public protein databases; Pfam (Pfam Protein families), Uniprot (Swiss-Prot). It comprised RSEM (v1.2.6) for transcript abundance estimation and normalization of expression values as FPKM (Fragments per kilobase of transcript per million fragments mapped). Differentially expressed genes were identified with DESeq2 with a filter threshold of *P*-value <0.05 and |log_2_FoldChange| > 1. ClusterProfiler(http://www.bioconductor.org/packages/release/bioc/html/clusterProfiler.html) in R package was employed to perform GO and KEGG (Kyoto Encyclopedia of Genes and Genomes, http://www.genome.jp/kegg/) enrichment analysis. The GO and KEGG enrichment analysis were calculated using hypergeometric distribution with a Q value cutoff of 0.05. Q values obtained by Fisher's exact test were adjusted with FDR for multiple comparisons.

*Synergistic potential of limonene formulation with chemical fungicides against Fusarium*

The EC_50th_ values were calculated according to the following equation:

EC_50th_ = (a + b) / [a / EC_50_ (A) + b / EC_50_ (B)]

Where ‘‘a” is the concentration of A in a mixture and ‘‘b” is the concentration of B in a mixture. EC_50_ (A) is the observed EC_50_ value of A, and EC_50_ (B) is the observed EC_50_ value of B.

The EC_50ob_ value was estimated via linear regression of the probit-transformed relative inhibition value (1-RG) at the log10 transformed-mixture concentration. The interaction level (R) of the mixtures was determined using the following equation:

R = EC_50th_ / EC_50ob_

The synergistic interaction of mixtures was defined as synergistic when R > 1.5, additive when 1.5 > R > 0.5, and antagonistic when R < 0.5.

**2. Supplementary tables**

**Table S1** The primer and information of the gene used in the RT-PCR.

| GeneName  Gene ID | Primer | Sequence(5’-3’) |
| --- | --- | --- |
| *FgTRI1*  FGSG_00071 | Tri1-F | CACAAAGCTAGACCAGTAG |
|  | Tri1-R | TATCGTTATCTTCACATGCT |
| *FgTRI4*  FGSG_03535 | Tri4-F | ACCAGGTCCTCAGTCTTG |
|  | Tri4-R | TCGTTGTGCTTGCCATAG |
| *FgTRI5*  FGSG_03537 | Tri5-F | TGAGGGATGTTGGATTGAGCAGTAC |
|  | Tri5-R | TGCTTCCGCTCATCAAACAGGT |
| *FgTRI6*  FGSG_03536 | Tri6-F | GCTACTCAGAATGCCCTCAG |
|  | Tri6-R | CGCATGTTATCCACCCTGCTA |
| *FgTRI12*  FGSG_02343 | Tri12-F | GCTGTAACTGTCCCCAGCAT |
|  | Tri12-R | GTGAAGTTGCGACCGTACTC |
| *FgTRI101*  FGSG_07896 | Tri101-F | ATACCCTATGGCGATGTTTGAC |
|  | Tri101-R | CTGTCCGTTGACAGTGAGGAT |
| *FgACTIN*  FGSG_07335 | Actin-F | ATCCACGTCACCACTTTCAA |
|  | Actin-R | TGCTTGGAGATCCACATTTG |

**Table S2** The EC_50_ of limonene formulation, tebuconazole, mefentrifluconazole and phenamacril against *F. graminearum* and *F. fujikuroi* (mg/L).

| Fungal strain | Limonene formulation | Tebuconazole | Mefentrifluconazole | Phenamacril |
| --- | --- | --- | --- | --- |
| *F. graminearum* | 70.0  (1.40 μL/mL) | 0.25 | 1.10 | 0.23 |
| *F. fujikuroi* | 40.0  (0.80 μL/mL) | 0.070 | 0.25 | 0.25 |

**Table S3** The detailed of the ratio and concentration gradient of limonene formulation in combination with chemical fungicides against *Fusarium*

| Fungal strain | *F. graminearum* | | | *F. fujikuroi* | | |
| --- | --- | --- | --- | --- | --- | --- |
| Ratio  Concentration(mg/L) | 1:1 | 2:1 | 5:1 | 1:1 | 2:1 | 5:1 |
| Limonene formulation: Tebuconazole | 0.10 | 0.030 | 0.10 | 0.033 | 0.030 | 0.10 |
|  | 0.20 | 0.20 | 0.20 | 0.10 | 0.10 | 0.20 |
|  | 0.40 | 0.40 | 0.40 | 0.20 | 0.20 | 0.30 |
|  | 0.60 | 2.0 | 0.80 | 0.30 | 0.30 | 0.40 |
|  | 1.20 | 3.0 | 1.60 | 0.40 | 0.40 | 0.60 |
| Limonene formulation: Mefentrifluconazole | 0.20 | 0.20 | 0.30 | 0.20 | 0.30 | 0.30 |
|  | 0.30 | 0.30 | 0.50 | 0.30 | 0.40 | 0.50 |
|  | 0.40 | 0.40 | 1.0 | 0.40 | 0.60 | 1.0 |
|  | 0.60 | 0.60 | 1.7 | 0.60 | 0.80 | 1.7 |
|  | 0.80 | 1.1 | 2.2 | 0.80 | 1.1 | 2.2 |
| Limonene formulation: Phenamacril | 0.40 | 1.0 | 1.5 | 0.2 | 0.17 | 1.5 |
|  | 1.0 | 2.0 | 3.0 | 0.4 | 1.0 | 2.0 |
|  | 2.0 | 3.0 | 5.0 | 1.0 | 2.0 | 3.0 |
|  | 3.0 | 4.0 | 7.0 | 2.0 | 3.0 | 5.0 |
|  | 4.0 | 5.0 | 9.0 | 3.0 | 4.0 | 7.0 |

**Table S4** The top 20 pathways and DEGs in the down-regulated KEGG identified in *F. graminearum* (*P*-value <0.05).

| Pathway | *P*-value | *P*.adjust | DEGs number |
| --- | --- | --- | --- |
| Glycolysis / Gluconeogenesis | 1.39E-06 | 8.54E-05 | 22 |
| Galactose metabolism | 1.88E-06 | 8.54E-05 | 16 |
| Glyoxylate and dicarboxylate metabolism | 2.64E-06 | 8.54E-05 | 19 |
| Methane metabolism | 1.91E-05 | 0.000462 | 13 |
| Nitrogen metabolism | 7.96E-05 | 0.001545 | 10 |
| Starch and sucrose metabolism | 0.000294 | 0.004748 | 19 |
| Tryptophan metabolism | 0.000398 | 0.005515 | 18 |
| Alanine, aspartate and glutamate metabolism | 0.001799 | 0.021791 | 13 |
| One carbon pool by folate | 0.002022 | 0.021791 | 7 |
| Cyanoamino acid metabolism | 0.004399 | 0.040477 | 10 |
| Sulfur metabolism | 0.004599 | 0.040477 | 9 |
| Amino sugar and nucleotide sugar metabolism | 0.005007 | 0.040477 | 18 |
| Pyruvate metabolism | 0.00603 | 0.044822 | 15 |
| Glycine, serine and threonine metabolism | 0.006931 | 0.044822 | 16 |
| Arginine and proline metabolism | 0.006931 | 0.044822 | 16 |
| Atrazine degradation | 0.013568 | 0.082253 | 3 |
| Vitamin B6 metabolism | 0.014777 | 0.084316 | 4 |
| Pentose and glucuronate interconversions | 0.020952 | 0.106965 | 10 |
| Fructose and mannose metabolism | 0.020952 | 0.106965 | 10 |
| Tyrosine metabolism | 0.038267 | 0.174768 | 14 |

**Table S5** The pathways and DEGs in the up-regulated KEGG identified in *F. graminearum* (*P*-value <0.05).

| Pathway | *P*-value | *P*.adjust | DEGs number |
| --- | --- | --- | --- |
| Ribosome biogenesis in eukaryotes | 6.76E-15 | 6.96E-13 | 42 |
| DNA replication | 3.67E-12 | 1.89E-10 | 25 |
| Citrate cycle (TCA cycle) | 3.10E-07 | 1.07E-05 | 19 |
| Ribosome | 6.84E-06 | 0.000176 | 41 |
| RNA polymerase | 1.90E-05 | 0.000392 | 15 |
| Mismatch repair | 3.29E-05 | 0.000565 | 13 |
| RNA transport | 0.000188 | 0.002773 | 32 |
| Cell cycle - yeast | 0.000355 | 0.004567 | 32 |
| Meiosis - yeast | 0.002274 | 0.02603 | 25 |
| Base excision repair | 0.003118 | 0.032112 | 11 |
| Biosynthesis of unsaturated fatty acids | 0.006065 | 0.056795 | 7 |
| ABC transporters | 0.013302 | 0.114177 | 9 |
| Steroid biosynthesis | 0.018677 | 0.147979 | 11 |
| Glycerophospholipid metabolism | 0.034171 | 0.251404 | 15 |

**Table S6** The synergistic potential of limonene formulation in combination with chemical fungicides against *F. graminearum*.

| Mixture | Ratio | EC_50ob_ ^a^  (μg/mL) | EC_50th_ ^b^  (μg/mL) | Virulence regression equation | R^2^ | Interaction level（R） | Synergistic interaction |
| --- | --- | --- | --- | --- | --- | --- | --- |
| LM: Teb | 1:1 | 0.167 | 0.498 | Y = 52.98x + 91.19 | 0.976 | 2.984 | Synergistic |
|  | 2:1 | 0.758 | 0.745 | Y = 25.11x + 53.02 | 0.916 | 0.982 | Additive |
|  | 5:1 | 0.423 | 1.474 | Y = 42.84x + 66.02 | 0.992 | 3.486 | Synergistic |
| LM: Mef | 1:1 | 1.171 | 2.166 | Y = 52.15x + 46.43 | 0.913 | 1.850 | Synergistic |
|  | 2:1 | 2.431 | 3.199 | Y = 62.96x + 25.71 | 0.958 | 1.316 | Additive |
|  | 5:1 | 3.967 | 6.119 | Y = 58.07x + 15.24 | 0.936 | 1.542 | Synergistic |
| LM: Phe | 1:1 | 0.361 | 0.458 | Y = 93.55x + 91.42 | 0.987 | 1.271 | Additive |
|  | 2:1 | 0.643 | 0.686 | Y = 58.09x + 61.15 | 0.972 | 1.066 | Additive |
|  | 5:1 | 1.358 | 1.345 | Y = 63.40x + 41.84 | 0.984 | 1.009 | Additive |

Note: ^a^EC_50ob_ value is the observed EC_50_, which was estimated by the linear regression of the probit-transformed relative inhibition value (1-RG) at the log10 transformed-mixture concentration. ^b^EC_50th_ value is the theoretical EC_50_, which was calculated following Wadley’s model. LM: Teb = Limonene formulation: Tebuconazole; LM: Mef = Limonene formulation: Mefentrifluconazole; LM: Phe = Limonene formulation: Phenamacril

**Table S7** The synergistic potential of limonene formulation in combination with chemical fungicides against *F. fujikuroi*.

| Mixture | Ratio | EC_50ob_^a^  (μg/mL) | EC_50th_ ^b^  (μg/mL) | Virulence regression equation | R^2^ | Interaction level ( R) | Synergistic interaction |
| --- | --- | --- | --- | --- | --- | --- | --- |
| Limonene formulation: Tebuconazole | 1:1 | 0.0966 | 0.1398 | Y = 42.207x + 92.847 | 0.9691 | 1.4472 | Additive |
|  | 2:1 | 0.1541 | 0.2093 | Y = 39.952x + 82.448 | 0.9903 | 1.3581 | Additive |
|  | 5:1 | 0.2724 | 0.4164 | Y = 46.083x + 76.029 | 0.9922 | 1.5288 | Synergistic |
| Limonene formulation: Mefentrifluconazole | 1:1 | 0.4827 | 0.4969 | Y = 64.65x + 70.45 | 0.9610 | 1.0294 | Additive |
|  | 2:1 | 0.6794 | 0.7407 | Y = 48.121x + 58.077 | 0.9873 | 1.0902 | Additive |
|  | 5:1 | 1.8168 | 1.4545 | Y = 68.554x + 32.223 | 0.9081 | 0.8006 | Additive |
| Limonene formulation: Phenamacril | 1:1 | 0.3959 | 0.4969 | Y = 106.07x + 92.688 | 0.9321 | 1.2552 | Additive |
|  | 2:1 | 0.6255 | 0.7407 | Y = 96.651x + 69.489 | 0.9933 | 1.1841 | Additive |
|  | 5:1 | 1.2278 | 1.4545 | Y = 79.01x + 42.957 | 0.9779 | 1.1846 | Additive |

Note: ^a^EC_50ob_ value is the observed EC_50_, which was estimated by the linear regression of the probit-transformed relative inhibition value (1-RG) at the log10 transformed-mixture concentration. ^b^EC_50th_ value is the theoretical EC_50_, which was calculated following Wadley’s model.

**3. Supplementary figures**


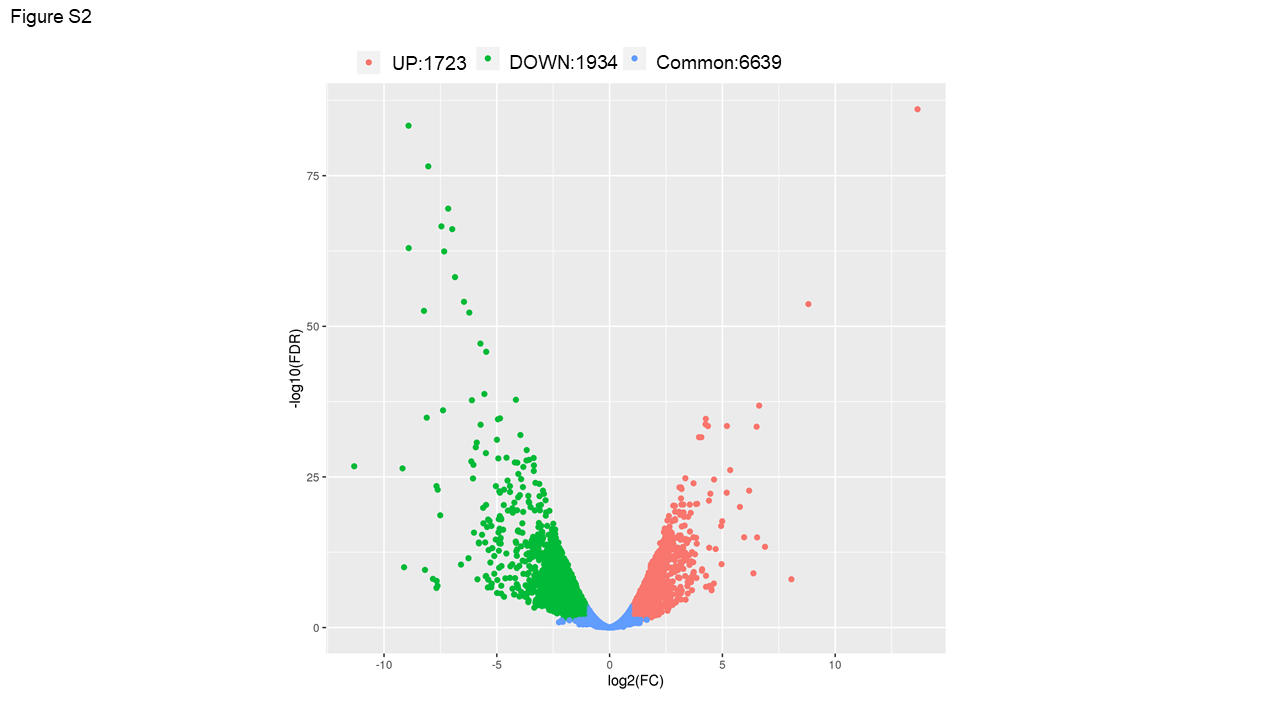


**FIGURE S1 |** The differentially expressed genes caused by 0.20 μL/mL limonene formulation in *F.graminearum*. Each point represents a gene. The green represented the down-regulated genes, the red represented the up-regulated genes, and the blue dots represented genes that have no significant differences.


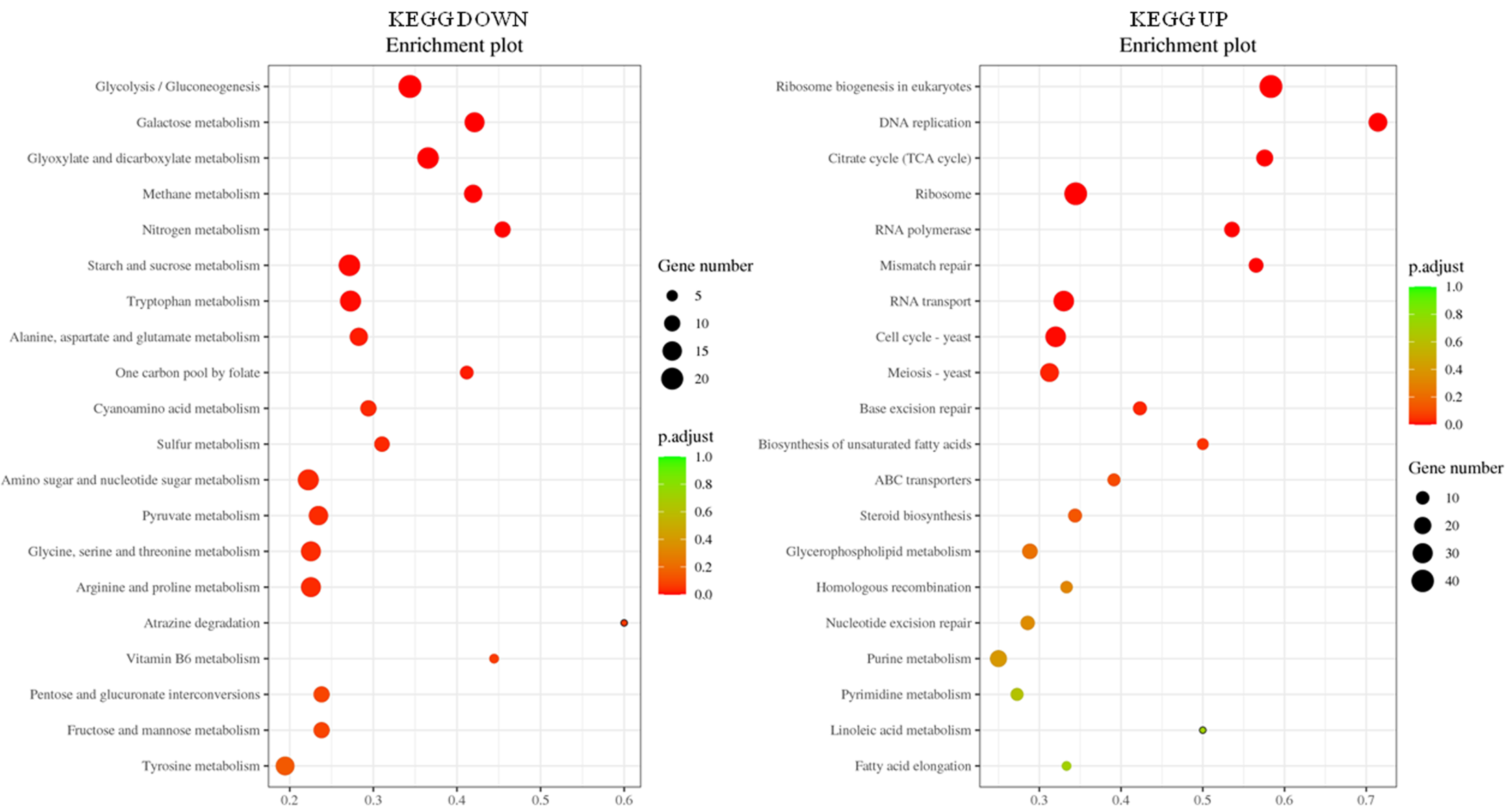


**FIGURE S2 |** The top 20 enriched KEGG pathways identified in *F. graminearum* after 0.20 μL/mL limonene formulation treatment.

**
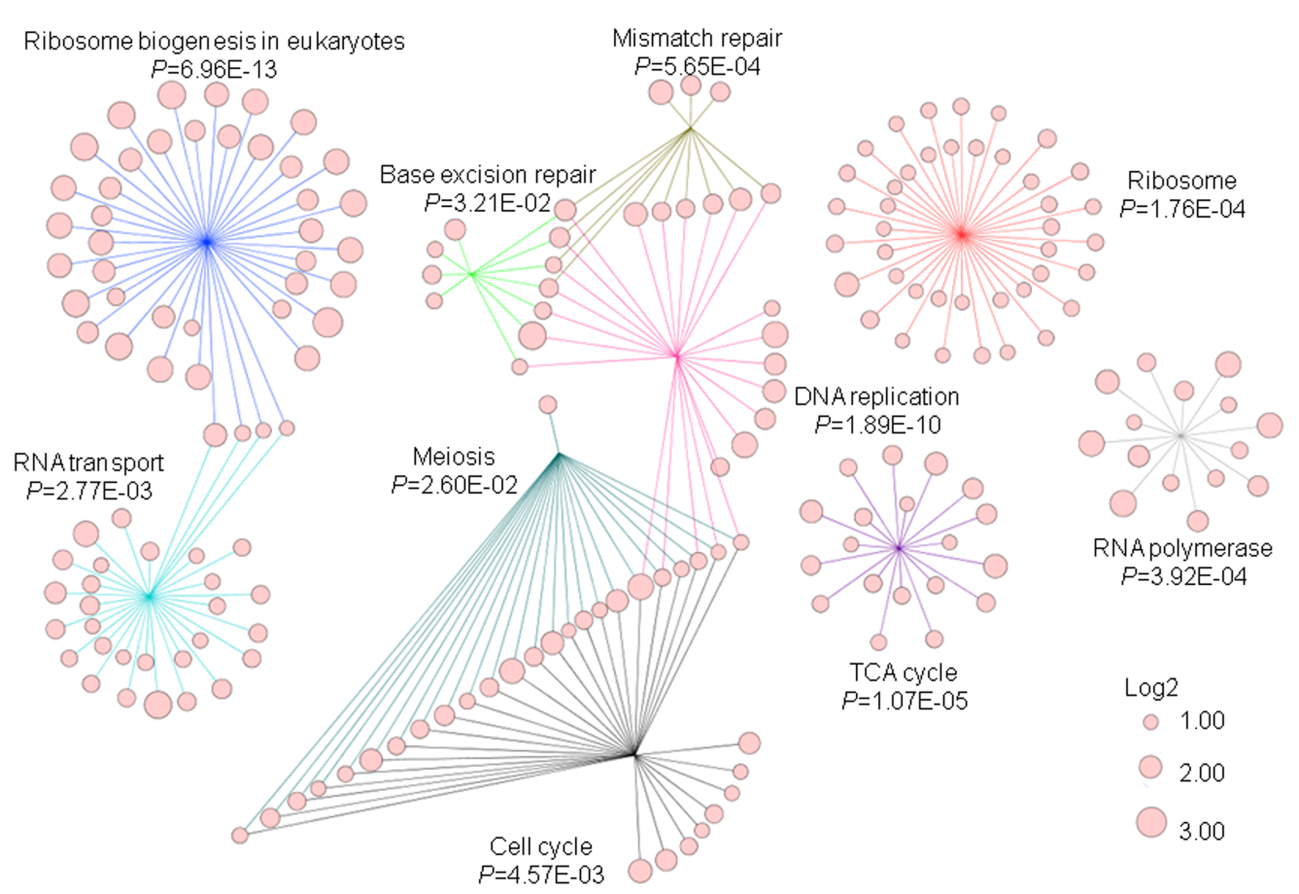
**

**FIGURE S3 |** Network analysis of the relationship between significantly up-regulated genes and corresponding KEGG pathways induced by limonene formulation.


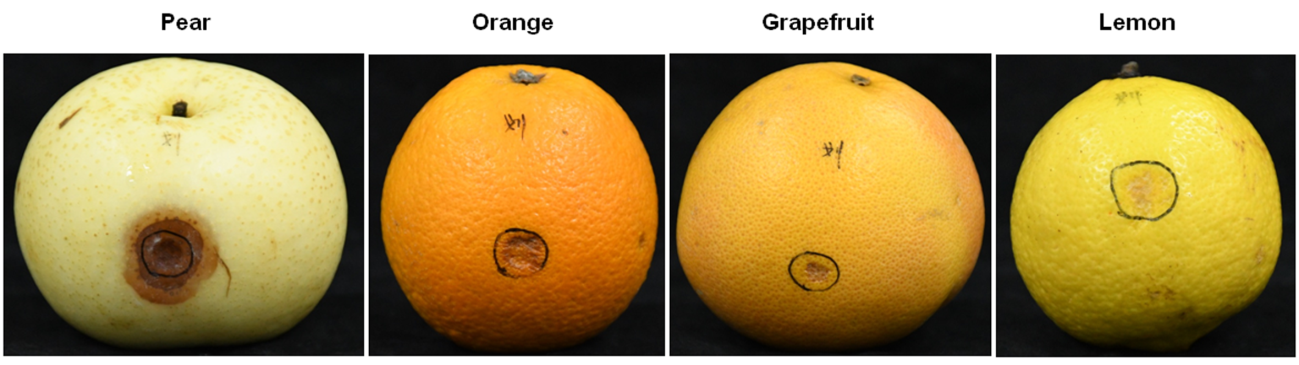


**FIGURE S4 |** D-limonene-containing fruits were effective in increasing resistance to *F.graminearum*. The representative fruits of Rutaceae were inoculated with mycelial plugs and examined 4 day after inoculated. Rosaceae pear was inoculated in same way as the positive control.
